# Attraction of *Chrysotropia ciliata* (Neuroptera, Chrysopidae) Males to P-Anisaldehyde, a Compound with Presumed Pheromone Function

**DOI:** 10.1007/s10886-020-01191-5

**Published:** 2020-06-26

**Authors:** Gunda Thöming, Sándor Koczor, Ferenc Szentkirályi, Hans R. Norli, Marco Tasin, Geir K. Knudsen

**Affiliations:** 1 Norwegian Institute of Bioeconomy Research, Division of Biotechnology and Plant Health, Box 115, NO-1431 Ås, PO Norway; 2 Plant Protection Institute, Centre for Agricultural Research, Budapest, Hungary; 3 Department of Plant Protection Biology, Swedish University of Agricultural Sciences, Alnarp, Sweden

**Keywords:** Green lacewing, Field trapping, Male-produced pheromone, Methyl p-anisate, P-methoxybenzoic acid

## Abstract

In a field-trapping experiment with plant volatiles, we observed notably high attraction of green lacewing (*Chrysotropia ciliata*) males to the compound p-anisaldehyde. Based on this finding, we initiated the present study to elucidate this phenomenon and to investigate the chemical ecology of *C. ciliata.* Scanning electron microscopy revealed elliptical glands abundantly distributed on the 2nd to 6th abdominal sternites of *C. ciliata* males, whereas females of the species completely lacked such glands. No p-anisaldehyde was found in extractions of body parts of *C. ciliata.* Methyl p-anisate and p-methoxybenzoic acid were identified exclusively in the extract from abdominal segments 2–8 of males. Field-trapping experiments revealed no attraction of *C. ciliata* to either methyl p-anisate or p-methoxybenzoic acid. In contrast, males showed marked attraction to p-anisaldehyde in the field and antennae showed strong responses to this compound. Headspace collections in the field from living insects in their natural environment and during their main daily activity period indicated that p-anisaldehyde was emitted exclusively by *C. ciliata* males. Our overall results suggest that p-anisaldehyde might serve as a male-produced pheromone that attracts conspecific *C. ciliata* males. Here, we discuss hypotheses regarding possible mechanisms involved in regulation of p-anisaldehyde production, including involvement of the compounds methyl p-anisate and p-methoxybenzoic acid, and the potential ecological function of p-anisaldehyde in *C. ciliata*.

## Introduction

The ability of conspecific insects to identify and find each other in the environment is determined by the power and effectiveness of intraspecific communication. Such exchange of information involves highly diverse mechanisms, systems, and tactics, which are chemical, visual, or acoustical in nature (Haynes and Yeargan 1999; Johansson and Jones 2007; Virant-Doberlet and Čokl 2004). It has long been known that lacewings (Neuroptera: Chrysopidae) communicate acoustically by producing low-frequency, substrate-borne vibrations to find mates located on plants (Duelli 1999; Henry 1979, 1980), and these insects have developed a sophisticated duetting behaviour to exchange these intraspecific signals (Henry and Wells 2004; Henry et al. 2012, 2013). Thus far, relatively little has been discovered about how lacewings achieve intraspecific communication via semiochemicals (Aldrich and Zhang 2016), although it has been reported that pheromones and cuticular hydrocarbons are involved in mate finding of some Chrysopidae species (reviewed by Aldrich and Zhang 2016; Henry et al. 2013). An adjustment of vibrational communication by additional communication via pheromones and vice versa or an interaction between both types of communication have also been shown for other insect species, such as *Nezara viridula* L. (Hemiptera: Pentatomidae) (Cocraft and Rodriguez 2005; De Groot et al. 2011; Virant-Doberlet and Čokl 2004). The first intraspecific attractants to be identified in green lacewings were the male-produced pheromone component (1*R*,2*S*,5*R*,8*R*)-iridodial in *Chrysopa oculata* Say (molecular weight 168; Zhang et al. 2004) and a closely related male-specific active compound in *C. nigricornis* Burmeister with the molecular weight 170 (Zhang et al. 2006a; Jeffrey R. Aldrich, University of California, US, pers. comm.), and, interestingly, for both those *Chrysopa* species, only males were caught in traps baited with the identified male pheromones. A compound of the defensive secretion, (*Z*)-4-tridecene, also elicited behavioural response, avoidance, from green lacewings, thereby having intraspecific effects as well (Koczor et al. 2018; Zhu et al. 2000).

In addition to intraspecific chemical signals, research has revealed diverse and subtle chemical relationships between lacewings and the environment (Szentkirályi 2001a), and several allelochemicals (interspecific chemical signals) affecting the behavioural ecology of green lacewings have been identified (reviewed by Aldrich and Zhang 2016). For example, recent studies have shown that a blend of common floral volatiles and herbivore-induced plant volatiles (methyl salicylate, acetic acid, and phenylacetaldehyde) acts as a strong attractant for *Chrysoperla carnea* s.l. (Stephens), and this attraction applies to both sexes*,* increasing egg laying in this species and thereby also augmenting aphid predation (e.g., Jones et al. 2016; Koczor et al. 2015; Pålsson et al. 2019; Tóth et al. 2009).

The green lacewing *Chrysotropia ciliata* Wesmael is widely distributed throughout the Palaearctic region. In particular, it is found in areas with broad-leaf forests in Western and Central Europe, Fennoscandia, parts of the Middle East, and Central and East Asia, including the Russian Far East and Japan. In southern Europe, the species occurs sporadically in the Mediterranean area; in northern Europe, its area extends to 60 degrees latitude (Aspöck et al. 1980, 2001; Makarkin 1985). *C. ciliata* is a stenotopic and strongly hygrophilous species that prefers habitats with cooler, shady, and humid microclimates, which are found in various types of temperate forests, such as deciduous broad-leaf forests, forests alongside creeks, forest clearings with rich vegetation, and planar or submontane floodplain gallery forests (Aspöck et al. 1980; Czechowska 1997; Gepp 1977; Gruppe 2007; **Makarkin** and **Shchurov**^2019^). In Switzerland, *C. ciliata* has been found to be a typical shrub belt inhabitant at structured forest edges (Duelli et al. 2002). *C. ciliata* is most commonly associated with trees of the species *Quercus*, mainly *Q. robur* and *Q. petraea* (Gruppe and Schubert 2001; Gruppe 2008; **Makarkin and Ruchin**^2019^**;** Monserrat and Marin 1994, 2001; Szentkirályi 2001c). Other important forest trees found to be inhabited by this chrysopid are *Fagus* spp., *Tilia cordata*, *Alnus glutinosa*, *Acer* spp., *Carpinus betulus*, *Ulmus* spp., and *Fraxinus* spp. (Czechowska 1997; Gruppe 2007, 2008; Gruppe and Schubert 2001; Nielsen 1977; Szentkirályi 2001c). Some sporadic incidence of *C. ciliata* has also been observed in cultivated areas, such as apple, walnut, and grape orchards (Pantaleoni and Alma 2001; Szentkirályi 2001b). Regular appearance of *C. ciliata* has been noted only in hazelnut plantations, which are characterized by a more humid microclimate (Monserrat and Marín 1994, 2001; Szentkirályi 2001b).

In Europe, it has been reported that the seasonal activity period of *C. ciliata* adults ranges from the end of April to late September, and that mass occurrence of this species can be noted in July and August (Aspöck et al. 1980; Duelli et al. 2002; Gepp 1977; Nielsen 1977; Saure and Kielhorn 1993; Szentkirályi 2001c). *C. ciliata* adults have a non-predatory, glycophagous diet that includes insect honeydew and nectars, or various plant exudates (Canard et al. 1990). The females lay eggs singly on leaves or branches in the crowns of trees, where the larvae subsequently develop on the underside of leaves. *C. ciliata* is a nocturnal species. The adults exhibit the ‘carnea’ type activity pattern, which is characterized by maximum activity during the first half of the night (Ábrahám and Vas 1999). The predatory larvae are debris carriers, with almost the entire dorsal area of their body covered with a shield made of prey skins and particles of lichens and plants (Diaz-Aranda and Monserrat 1995; Gepp 1983, 1987; New 1969). Although few studies have examined predation by *C. ciliata* larvae, there is evidence that the main types of prey probably include aphids, coccids, psyllids, and mealy bugs infesting tree branches and leaves (Nielsen 1977; Szentkirályi 2001c). Considering that *C. ciliata* has been detected in large numbers at canopy level in several forest areas (Czechowska 1997; Gepp 1977; Gruppe 2007; Gruppe and Schubert 2001; New 1969; **Makarkin and Ruchin**^2019^), it is possible that this chrysopid species can be an important biological control agent of homopteran pests in Palaearctic deciduous forest zones.

In a field screening of the use of *Sorbus aucuparia* plant volatiles as insect attractants that we conducted in Sweden in 2003, we recorded high catches of the green lacewing *C. ciliata* in p-anisaldehyde-baited traps, and all of the captured individuals were males. Based on this initial observation and on the findings mentioned above, we initiated the present study to better elucidate the chemical ecology of *C. ciliata*, with a particular focus on intraspecific communication.

## Methods and Materials

### Insects

For scanning electron microscopy (SEM) and chemical analyses, we collected *C. ciliata* in Norway in old mixed forests near small creeks in Ås, Akershus County, and Hobøl, Østfold County, during the period June–August in 2016 and 2018. Different trapping methods were used for males and females to collect individuals for further analyses as males but no females of *C. ciliata* were trapped at ground level of the trees. Males were collected by sweep netting in the evening or early morning in forest vegetation at a height of 0.5–1.5 m. Females were captured at night by using light traps (Safari® moth trap, JoTech Ltd., t/a Insectopia, Austrey, UK) equipped with a multi-tube 20 W BL368 light bulb (Casell Lighting) and hung at a height of 7–10 m in old deciduous trees. For electrophysiological analyses, *C. ciliata* males were collected in a riverside forest near Tököl, Hungary, using p-anisaldehyde-baited traps and by sweep netting.

### Scanning Electron Microscopy

Male and female *C. ciliata* were frozen at −80 °C directly after sampling. Legs and wings were removed from the frozen insects (five males and three females), and thereafter the lacewings were mounted (ventral and dorsal position, respectively) on brass stubs and coated with gold/palladium using a Polaron Sputter Coater (SC 7640, UK). The coated specimens were examined in a Zeiss EVO-50-EP scanning electron microscope (Carl Zeiss AG, Oberkochen, Germany) at an accelerating voltage of 10.0 kV.

### Extract Preparations and Volatile Collections in the Field

Adults of both sexes were kept in insect cages (30 × 30 × 30 cm; BugDorm-1, Mega View Science, Taichung, Taiwan) in the laboratory, providing water for the insects on moist cotton balls. For extracts all insects were dissected within 2–15 h of capture. Eight males and four females were anaesthetized by placing them in a freezer at −18 °C for 2–3 min, and body parts were obtained as cuticle that was dissected and dried with tissue paper. The parts were chosen and extracted individually in 50 μl methyl tert-butyl ether (MTBE), according the protocol outlined by Zhang et al. (2004): abdominal tip (last segment), abdominal cuticle (segments 2–8), and thorax. The extracts were stored at −80 °C pending use in chemical analyses.

For field headspace collection, *C. ciliata* males and females were collected in mixed forests near small creeks, and were subsequently kept at the collection sites in insect cages (30 × 30 × 30 cm) and subjected to analysis within 2–22 h. A cotton ball soaked with water was provided in each cage. Field headspace sampling was performed for 3 h, to collect the volatiles from live males and females (*N* = 3) during their main period of activity between 19:00 and 00:00. Volatiles were collected through a mobile air entrainment kit. Each adult *C. ciliata* was investigated in parallel with a blank control: the insect was carefully placed in a 250-ml glass chamber, and an identical but empty glass chamber served as the control. The bottoms of the two chambers were sealed with ground glass lids. Purified air was pumped into the bottom of the chambers through an inlet port of Teflon tubing at a rate of 30 ml min ^−1^, and the air was pumped through the filters at 25 ml min ^−1^. For technical specification of the setup of the mobile air entrainment kit and field headspace collections, see Steen et al. (2019). The volatiles emitted from the live adult were collected using Porapak Q polymer filters (35 mg, 80/100 mesh; Alltech, Deerfield, IL, USA) and eluted with 0.3 ml of hexane, as described by Thöming et al. (2014). Immediately thereafter, the samples were placed on dry ice and transported to the laboratory, where they were stored at −80 °C pending chemical analyses.

### Chemical Analyses

For chemical analyses, we used an Agilent 6890 N gas chromatograph connected to an Agilent 5973 mass spectrometer and an autosampler. The chromatograph was operated in splitless mode at 250 °C with an injection volume of 1 μl. We used a fused silica Agilent J & W Scientific DB-Wax separation column (Agilent Technologies; 30 m long, internal diameter 0.25 mm, film thickness 0.25 μm), and a 2.5-m methyl-deactivated precolumn (Varian Inc., Lake Forest, CA, USA) with the same internal diameter was connected to the analytical column via a press-fit connector (BGB Analytik AG, Boeckten, Switzerland). After injection of a sample, the temperature was held at 40 °C for 2 min and subsequently raised 6.9 °C/min to 160 °C and then 21.5 °C/min to 250 °C. Thereafter, the temperature was held constant at 250 °C for 3.6 min. The total running time was 27.18 min (Thöming et al. 2014). Volatile compounds were identified and quantified by combined gas chromatography and mass spectrometry (GC-MS) (Dalen et al. 2015). Volatile compounds were identified using Deconvolution Reporting Software (DRS, ver. A.03.0.84; Agilent Technologies, Santa Clara, CA, USA), which combines automatic MS deconvolution and identification software (AMDIS version 2.71, NIST) with an MS library (NIST05 database) and GC-MS software (ChemStation ver. D.03.00) (Agilent Technologies). The AMDIS database contained 1279 volatile compounds, 277 of which were connected to Kovats retention indices (Kovats 1958). To obtain comparable retention times for all samples, the retention time was locked and referenced according to the internal standard heptyl acetate at 10.75 min using the ChemStation retention time-locking program. Peaks that were present in the chromatogram but were not identified by the DRS were manually interpreted using the NIST05 database. To ensure reliable identification, a match factor of ≥70 was employed (Stein et al. 1999). Identification of compounds was verified by comparing mass spectra and Kovats indices with those obtained for synthetic standards on the same column. The compounds were acquired as standards from Sigma-Aldrich, SAFC, and Fluka.

In addition to extract preparations and volatile collections, we performed GC-MS to analyse synthetic compounds that attracted *C. ciliata* in the field-trapping experiments.

### Electrophysiological Analyses

In a preliminary screening, synthetic stimuli were tested on male *C. ciliata* antennae for electroantennographic (EAG) activity. A stimulus was delivered to an antenna through a stainless steel tube (Teflon-coated inner wall) with a constant humidified airflow. An antenna was used directly after being amputated at the base from a live lacewing and mounted between two glass capillary electrodes containing Ringer’s solution. The mounted antenna was placed at a distance of approximately 3 mm from the odour-delivering airflow. One of the electrodes was grounded, and the other was connected to a high-impedance DC amplifier (IDAC-2, Syntech, Kirchzarten, Germany). Synthetic compounds (5-μl load) were provided on a Rotilabo filter disc (diam. 10 mm; RKTech Kft., Budapest, Hungary) inside a Pasteur pipette. Synthetic standards of p-anisaldehyde, methyl salicylate, methyl p-anisate, 2-phenylethanol, and (*E*)-anethole were tested. (*Z*)-3-hexenol (5 μl of 1 μg/μl hexane solution) was used as a reference treatment, which was tested before and after the other stimuli, and against which response amplitudes were normalized. Solvent (hexane) and blank air were used as controls. Stimuli were presented by a Syntech CS-55 stimulus controller (Syntech, Kirchzarten, Germany). Pulse duration was 0.5 s, and stimuli were administered at approximately 20–30-s intervals. Recordings of the antennal responses to each compound were replicated 12 times, each time using a new antenna from another male.

### Field Trapping

#### Experiment 1

Based on the initial field screening of *Sorbus aucuparia* plant volatiles conducted in 2003, a trapping experiment targeting *C. ciliata* was set up in 2004. At each of two locations in southern Sweden, Alnarp and Sjöbo, five replicates of Tetra traps (PheroNet AB, Lund) were placed at a height of 1.5–2.0 m in deciduous trees with a minimum of 10 m between traps. The traps were placed at lower level of the canopy as preliminary trapping tests have shown that a solid number of males can be found at this level. The traps were installed on May 19 and were checked six times up to July 16. Red rubber septa (VWR International) baited with 2-phenylethanol (10 mg), anethole (10 mg), p-anisaldehyde (10 mg), 2-phenylethanol + p-anisaldehyde (10 + 10 mg), anethole + p-anisaldehyde (10 + 10 mg), and control (methanol solvent) were used as attractants. The Sjöbo location is a moist natural mixed forest, and the Alnarp location is a semi-natural park. No *C. ciliata* individuals were trapped at the Alnarp site, and hence the results shown are from the Sjöbo location.

Further field-trapping experiments designed for *C. ciliata* were carried out from 2016 to 2018 in Norway, Hungary, and Sweden in mixed forests located near small creeks or rivers.

#### Experiment 2

In Hungary, we conducted a dose-response experiment using CSALOMON® VARL+ funnel traps (produced by Plant Protection Institute, CAR, Budapest, Hungary). P-anisaldehyde was obtained from Sigma Aldrich Kft. (Budapest). Baits were prepared as follows: 1, 10, or 100 mg of p-anisaldehyde (≥ 98% purity according to the manufacturer) was applied on pieces of dental roll (Celluron®, Paul Hartmann AG, Heidenheim, Germany) placed in polyethylene vial dispensers with lids (No. 730, Kartell Co., Italy), after which the lids of the dispensers were closed. The PE vials were cylindrical, 32 mm long with 8 mm outer and 5.5 mm inner diameter and after deployed, the lids remained closed, to ensure slow diffusion through the dispenser walls. For easy handling, dispensers were attached to plastic handles (8 × 1 cm) and were wrapped separately in aluminium foil and stored at −18 °C until used. The following treatments were tested in the experiment: traps baited with 1, 10, or 100 mg of p-anisaldehyde and unbaited traps. The experiment was performed in 2016 from August 1 to October 24 in a riverside forest near Tököl using a randomized block design with six blocks. As a rule, the position of the traps was rotated every second week.

#### Experiment 3

Methyl salicylate has previously been found to have a synergistic effect on attraction of other green lacewing species (e.g., Tóth et al. 2009; Zhang et al. 2004). Inasmuch as methyl salicylate elicited antennal responses in the EAG screenings, we set up an experiment in Hungary to test methyl salicylate in combination with p-anisaldehyde. The trap design, dispenser, and experimental layout used were similar to those applied in Experiment 2. The load of individual compounds was kept at 50 mg. The following treatments were included: p-anisaldehyde alone, p-anisaldehyde plus methyl salicylate, and unbaited traps. The experiment was conducted in five blocks at the Tököl location in 2017 from May 23 to October 9. As a rule, the position of the traps was rotated every second week.

#### Experiment 4

A field-trapping experiment was performed at different locations in Norway, Hungary, and Sweden to investigate field attraction of *C. ciliata* to p-anisaldehyde, methyl p-anisate, and a control (Exp. 4A in 2017), and to p-anisaldehyde, p-methoxybenzoic acid, and a control (Exp. 4B in 2018).

In Norway, white delta traps with a sticky yellow base (9 × 16 × 9 cm; Silvandersson AB, Knäred, Sweden) were baited with 10 mg of the test compounds p-anisaldehyde (Fluka), methyl p-anisate (SAFC), and p-methoxybenzoic acid (Sigma–Aldrich), respectively, diluted in methanol (Sigma–Aldrich) and applied on a dispenser consisting of a cotton wick (length 1 cm; Parotisroll, Size 5, Roeko, Langenau, Germany) placed inside a polyethylene vial (1.5 ml; closed vial with approx. 0.2 mm hole in the lid; Easy-Fit, Treff, Degersheim, Switzerland). As controls, we used traps loaded with the solvent only. The traps were hung at a height of 1–1.5 m in deciduous trees. Single replicates of a set of respective test compounds (*N* = 6) were installed at each location in eastern Norway (three locations in Ås, Akershus County, and three in Hobøl, Østfold County) in 2017 and 2018. Traps were randomly spaced approximately 15 m apart. The trap positions were rotated within each replicate once a week. The insects that were caught were removed from the traps every 3–4 days, and sticky inserts and dispensers were replaced every second week. Experiments in 2017 (testing compounds p-anisaldehyde vs. methyl p-anisate vs. control) and 2018 (testing p-anisaldehyde vs. p-methoxybenzoic acid vs. control) were carried out in the same locations in eastern Norway. In 2018, using the same experimental treatments as above, 10 replicates were placed at a height of 1.5–2 m in deciduous trees in a mixed forest in southern Norway (Tromøy, Arendal, Agder County), and the traps were randomly spaced approximately 15 m apart and were checked twice between May 27 and July 24.

In Hungary, the trap design, dispenser, and experimental setup applied were similar to those used in Experiment 2. In both 2017 and 2018, the experiment was performed using randomized complete block design with five blocks. In 2017, the experiment was conducted from May 23 to October 9, and the treatments included p-anisaldehyde only, methyl p-anisate only, and unbaited traps. For both p-anisaldehyde and methyl p-anisate, 50 mg was loaded in the traps. In 2018, the experiment was performed from July 2 to October 4, and treatments included p-anisaldehyde only, p-methoxybenzoic acid only, and unbaited traps. For both p-anisaldehyde and p-methoxybenzoic acid, 10 mg was loaded in the traps. P-methoxybenzoic acid was dissolved in methanol. As a solvent control, we also set up methanol-baited traps, but they did not catch any lacewings and hence were omitted from further analyses.

In Sweden, a trapping test was carried out in 2018 in a broad-leaf forest along the Lödde River in Furulund from June 18 to July 9. This was done using the same trap material as described above for the experiments in Norway. Trap checking and sticky liner replacement were done at 7-day intervals. Sticky liners were brought to the laboratory and kept at −18 °C pending identification of *C. ciliata* males in a stereomicroscope.

## Statistics

For EAG data, treatments were compared by Kruskal-Wallis test followed by pairwise comparisons using the Wilcoxon rank sum test with Bonferroni correction. For field-trapping data, the numbers of insects captured were analysed using generalized linear mixed models (GLMMs) with a Poisson distribution (PROC GLIMMIX, SAS 9.4). The counts of *C. ciliata* males were used as the response variables, with considering treatment, location, and interaction between treatment and location as fixed factors. After establishing the significance of the fixed factors, Tukey’s tests were performed for pairwise comparisons between levels of each factor when necessary. For Experiments 2 and 3, catches were summed for trap rotation periods, and periods in which less than a total of five individuals were caught were excluded from the analysis. A significance level of α = 0.05 was applied in all analyses.

## Results

### Scanning Electron Microscopy

**Fig. 1:**
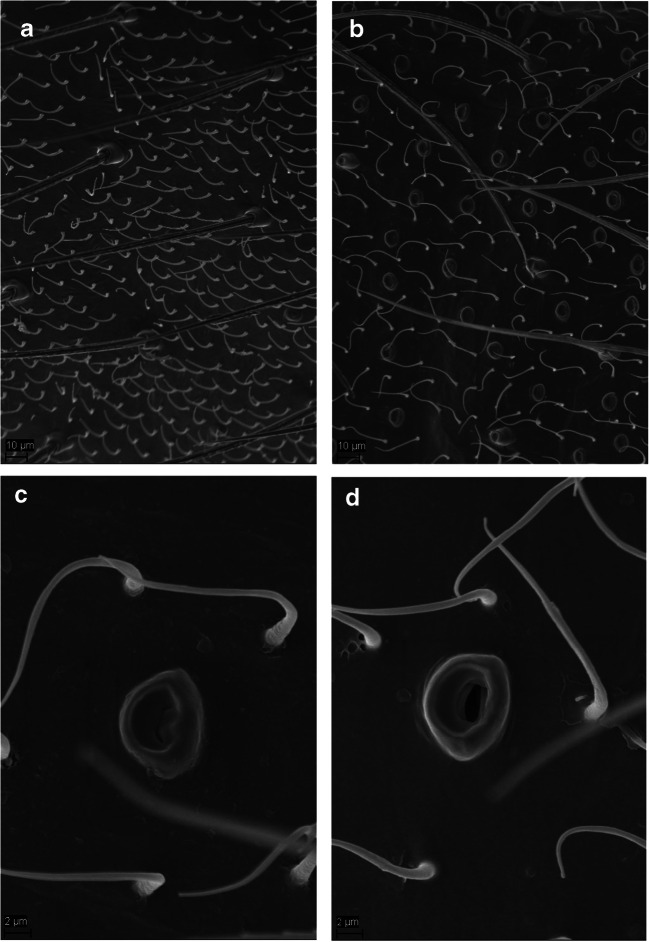
Scanning electron microscopy images of the cuticle of the 3rd abdominal sternite of a female (A) and a male (B) *C. ciliata*, and detailed images of a closed gland in the 3rd abdominal sternite (C) and an open gland in the 5th abdominal sternite (D) of a *C. ciliata* male

Elliptical epidermal glands (approx. 8 × 5.5 μm with a central slit) occurred on the cuticle of the 2nd to 6th abdominal sternites of *C. ciliata* males. These glands were not found on the remaining abdominal sternites or on the thorax of males. Females of *C. ciliata* completely lacked such glands (Fig. 1). We estimated that the males had approximately 10–13/100 μm^2^ of these epidermal glands, which were evenly distributed all over sternites 2–6.

### Chemical Identification

**Fig. 2:**
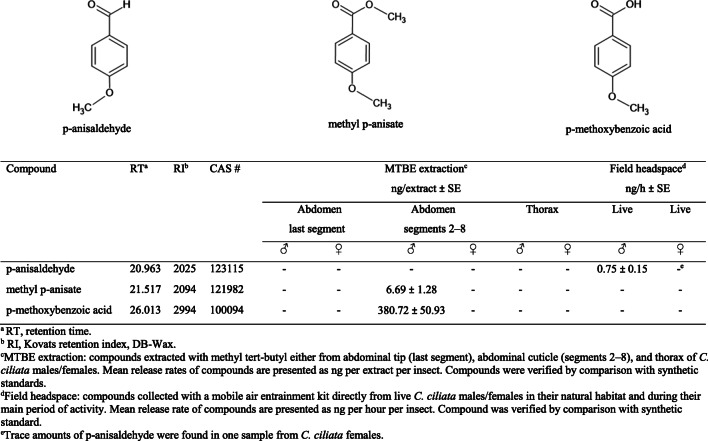
Volatile compounds identified and tested in the field regarding attraction of *C. ciliata* males. The diagram indicates the presence or absence of each compound in methyl tert-butyl ether (MTBE) extracts from samples of abdominal tip (last segment), abdominal cuticle (segments 2–8), and thorax of *C. ciliata* (males and females), and headspace collections performed in the field directly from live *C. ciliata* (males and females) in their natural habitat and during their main period of daily activity

No p-anisaldehyde was found in the MTBE extracts of the abdominal tip (last segment), abdominal cuticle (segments 2–8), or thorax of either male or female *C. ciliata*. The GC-MS analyses of MTBE extracts showed that methyl p-anisate and p-methoxybenzoic acid were present in all samples of abdominal cuticle (segments 2–8) from males, whereas these compounds were absent in extracts from both the abdominal tip (last segment) and thorax of males and in all samples (abdominal tip, abdominal cuticle segments 2–8, and thorax) from females (Fig. 2). By comparison, chemical analyses of headspace samples collected in the field from live insects showed p-anisaldehyde in males but not in females, although trace amounts of p-anisaldehyde were found in one sample from females. Methyl p-anisate and p-methoxybenzoic acid were not detected in any of the headspace samples that were analysed (Fig. 2).

### Antennal Recordings

**Fig. 3:**
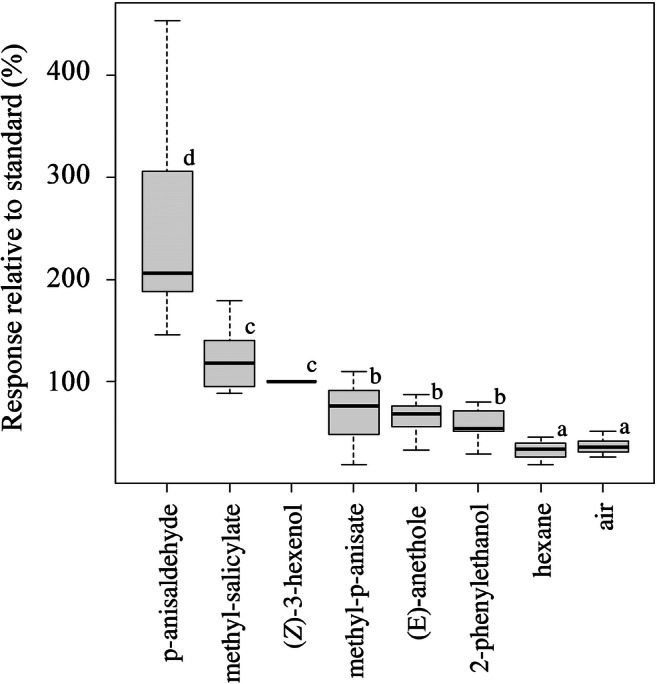
Electroantennographic responses (expressed as percentage relative to the standard) exhibited by *C. ciliata* males (*N* = 12) exposed to the synthetic standards of p-anisaldehyde, methyl salicylate, methyl p-anisate, 2-phenylethanol, and (*E*)-anethole. (*Z*)-3-hexenol was used as standard and was tested before and after the other stimuli. Hexane and blank air were tested as controls. Lower case letters indicate statistically significant differences (Kruskal-Wallis test, followed by pairwise comparisons using the Wilcoxon rank sum test with Bonferroni correction, *p* < 0.05)

The antennal responses of *C. ciliata* males are shown in Fig. 3. All compounds elicited significantly stronger responses compared to the controls (hexane or clean air). P-anisaldehyde induced the strongest response. The response to methyl salicylate did not differ significantly from the response to the reference (*Z*)-3-hexenol. Methyl p-anisate, 2-phenylethanol, and (*E*)-anethole elicited significantly lower responses compared to (*Z*)-3-hexenol (Fig. 3).

### Field-Trapping Experiments

**Fig. 4:**
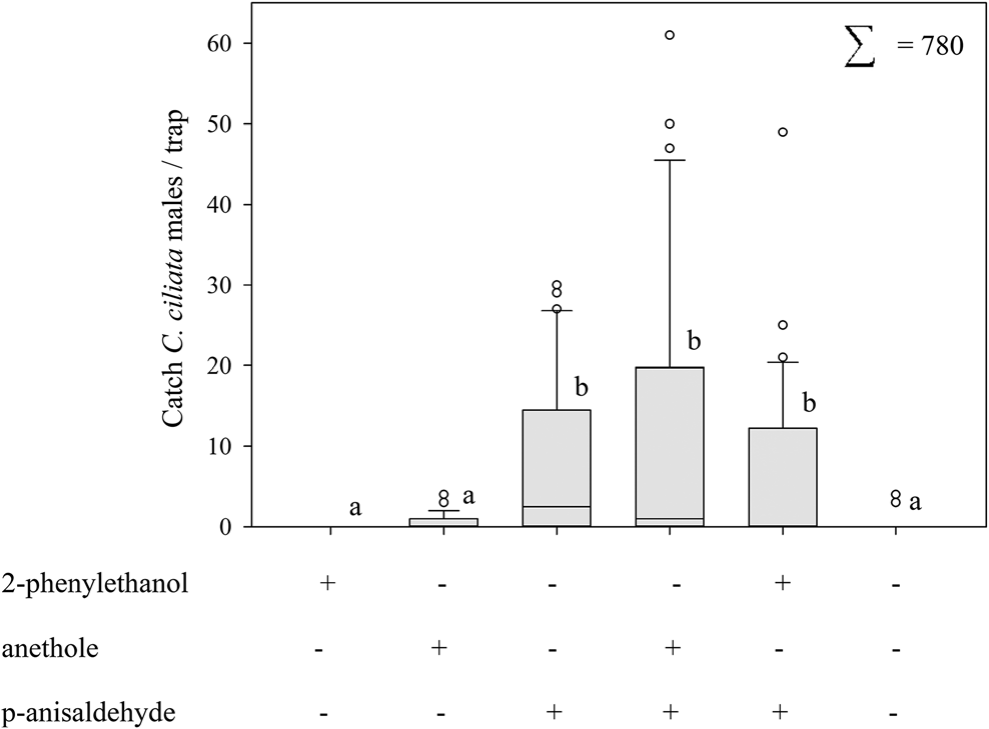
Trap catches (mean ± SE) of *C. ciliata* males in traps baited with candidate semiochemicals in Sjöbo in southern Sweden in 2004. The load of each compound was 10 mg. The results represent the cumulative value of six trap inspections over the period May 19 to July 16 *(N* = 5). Trap catches are presented as boxplots. The boxes (bars) representing the interquartile range, that is data within the 25th to 75th percentile and horizontal lines going through the boxes at the medians. Whiskers representing the data range to the minimum and maximum, that is data inside the 10th and 90th percentiles. Dots are the outliers, that are all data points that lie outside the 10th and 90th percentiles. Lower case letters indicate statistically significant differences (GLIMMIX, Tukey, *p* < 0.05)

In Experiment 1, 780 *C. ciliata* males were caught. The treatments applied in this experiment were the only factor found to elicit significant effects (GLMM: *F* = 7.49 _5,150_, *P* < 0.0001). Traps baited with p-anisaldehyde, a 1:1 blend of p-anisaldehyde and anethole, or a 1:1 blend of p-anisaldehyde and 2-phenylethanol caught significantly more males than traps loaded with anethole or, 2-phenylethanol separately, or the unbaited control traps (Fig. 4). Catches with the various treatments containing p-anisaldehyde did not differ significantly. Chemical analyses of anethole revealed traces of p-anisaldehyde.

**Fig. 5:**
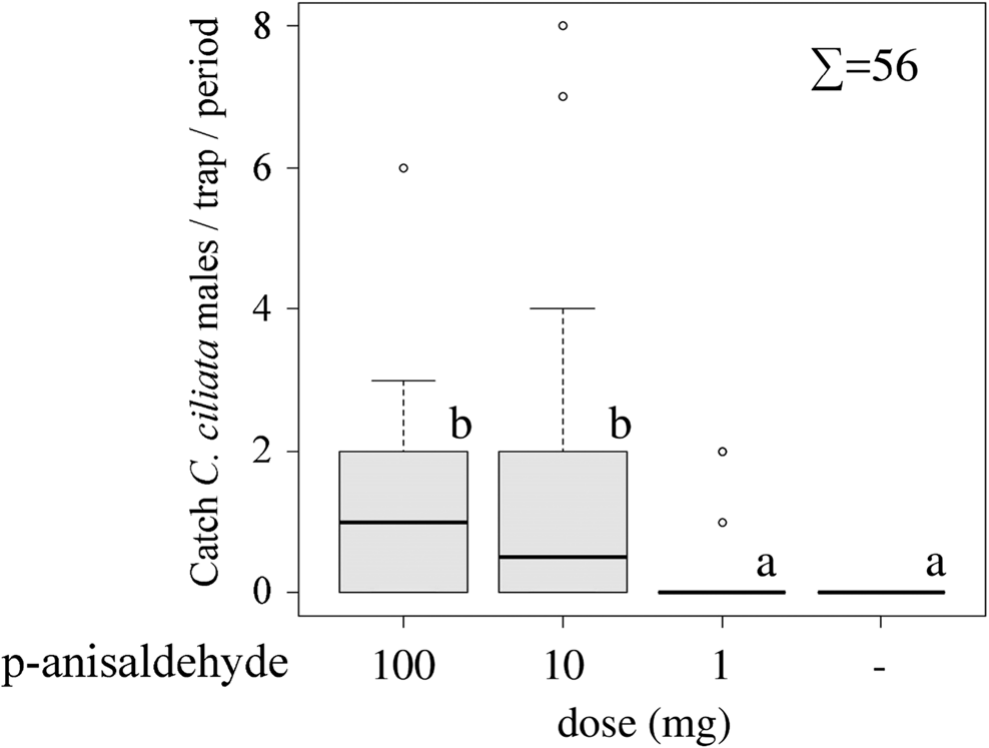
Trap catches (mean ± SE) of *C. ciliata* males in response to the three doses (100, 10, and 1 mg) of p-anisaldehyde and a control recorded in Tököl, Hungary, in 2016. As a rule, trap positions were rotated every second week, and catches are summed for trap rotation periods. Such periods during which less than five individuals were caught were excluded from the analysis. Trap catches are presented as boxplots (see legend of Fig. 4). Lower case letters indicate statistically significant differences (GLIMMIX, Tukey, *p* < 0.05)

In Experiment 2, 56 *C. ciliata* males were caught*.* As in experiment 1, the treatment was the only factor showing significant effects (GLMM: *F* = 4.80 _3,113_, *P* = 0.0035). *C. ciliata* males showed a positive dose response to p-anisaldehyde, as indicated by the greater number of individuals caught in traps loaded with higher doses of that compound (Fig. 5).

**Fig. 6:**
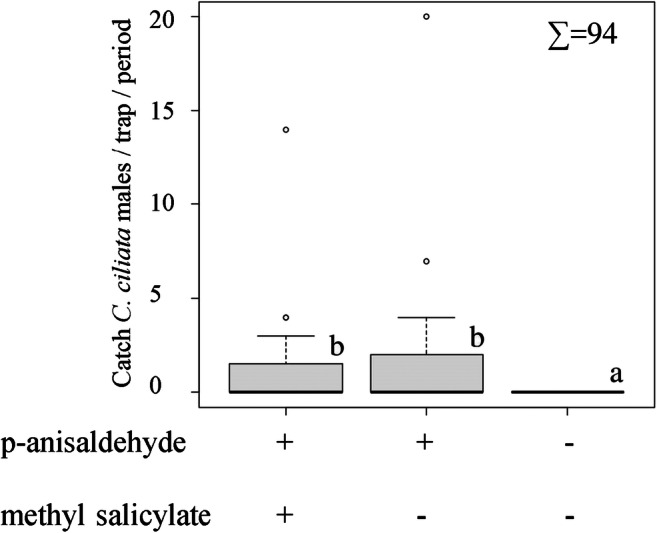
Trap catches (mean ± SE) of *C. ciliata* males in response to the two synthetic compounds p-anisaldehyde and methyl salicylate and a control recorded in Tököl, Hungary, in 2017. The load was 50 mg for each compound. As a rule, trap positions were rotated every second week, and catches are summed for trap rotation periods. Such periods during which less than five individuals were caught were excluded from the analysis. Trap catches are presented as boxplots (see legend of Fig. 4). Lower case letters indicate statistically significant differences (GLIMMIX, Tukey, *p* < 0.05)

In Experiment 3, a total of 94 *C. ciliata* males were captured. Again, treatment was the only factor showing significant effects (GLMM: *F* = 3.69 _2,118_, *P* = 0.0279). Traps baited with p-anisaldehyde and a 1:1 blend of p-anisaldehyde and methyl salicylate caught significantly more males compared to the control. However, methyl salicylate did not have a synergistic or additive effect when combined with p-anisaldehyde (Fig. 6).

**Fig. 7:**
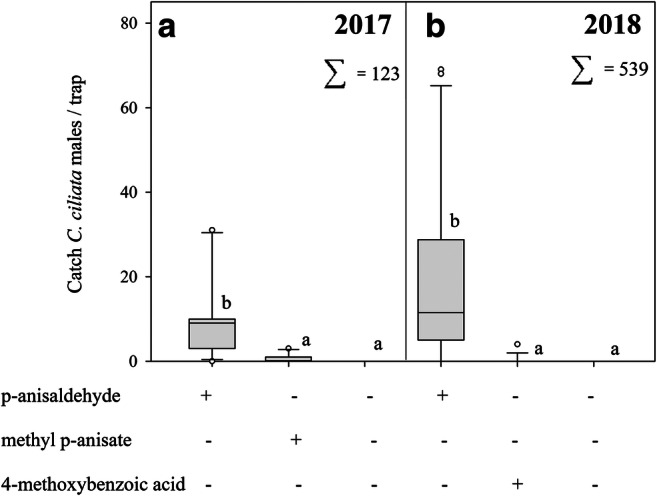
Catches (mean ± SE) of *C. ciliata* males in traps baited with p-anisaldehyde, methyl p-anisate, p-methoxybenzoic acid, or a control. The load of each compound was 10 mg. The results are the cumulative values recorded in eastern Norway (trap inspections over the period June 6–August 31*; N* = 6) and Hungary (trap inspections May 30–October 9; *N* = 5) in 2017 (A), and in eastern Norway (trap inspections June 6–July 31; *N* = 6), southern Norway (trap inspections May 27–July 24; *N* = 5), southern Sweden (trap inspections June 18–July 9; *N* = 5), and Hungary (trap inspections July 9–October 4; *N* = 5) in 2018 (B). Trap catches are presented as boxplots (see legend of Fig. 4). Lower case letters indicate statistically significant differences in 2017 and 2018 (GLIMMIX, Tukey, p < 0.05)

In Experiment 4, a total of 662 *C. ciliata* males were trapped as follows: in 2017, 63 in eastern Norway and 60 in Hungary; in 2018, 73 in eastern Norway, 394 in southern Norway, 42 in Hungary, and 30 in Sweden. In both years, location and interaction between location and treatment were not significant. The treatment was the only factor showing significant effects (GLMM_2017_: *F* = 9.75 _2,27_, *P* = 0.0006; GLMM_2018_: *F* = 21.48 _2,66_, *P* < 0.0001). In both years, nearly all *C. ciliata* males were caught in traps baited with p-anisaldehyde, although a few specimens were captured in traps baited with methyl p-anisate or 4-methoxybenzoic acid (Fig. 7). No *C. ciliata* females and neither significant numbers of nontarget species were caught in any of the field-trapping experiments (Figs. 4–7).

## Discussion

Our results suggest that p-anisaldehyde serves as a male-produced pheromone that attracts conspecific males of *C. ciliata*. Scanning electron micrographs revealed that the presumed pheromone is most likely released from elliptical glands that are abundantly distributed on the 2nd to 6th abdominal sternites of males, and which *C. ciliata* females completely lack. Other investigators (Zhang et al. 2004) have found that the green lacewing *Chrysopa oculata* has a similar pheromone system in which males release the pheromone (1R*,2S*,5R*,8R*)-iridodial to attract conspecific males, and they also exhibit an abundance of elliptical glands on the 3rd to 8th abdominal sternites. Furthermore, the authors proposed that the elliptical glands are responsible for production of the mentioned pheromone, because the abundance of the observed glands agreed with the detection of (1R*,2S*,5R*,8R*)-iridodial extracted from abdominal cuticle of segments 1–8 in males.

Our analyses revealed no p-anisaldehyde in extracts from body parts of *C. ciliata.* Instead, methyl p-anisate and p-methoxybenzoic acid were identified solely in the extracts from the 2nd to 8th abdominal segments of males. These findings led to our initial hypothesis that these two compounds might have pheromonal function. However, our experiments showed that methyl p-anisate and p-methoxybenzoic acid were not attractive to *C. ciliata* in the field. Furthermore, methyl p-anisate did not elicit significant responses in an EAG screening. In contrast, p-anisaldehyde was highly attractive to *C. ciliata* males in the field, and antennae of males exhibited a marked EAG response to this compound.

Our headspace collection in the field with living insects in their natural environment enabled us to confirm that p-anisaldehyde is released exclusively by males. It should be noted that this headspace sampling did reveal a trace amount of p-anisaldehyde in one live female. Still, this observation might have been the result of contact with a male *C. ciliata* immediately before the capture rather than the release of the compound by the female, although further experiments are needed to clarify this point. We hypothesize that, rather than being stored in a specific reservoir, p-anisaldehyde is released exclusively by males under specific conditions to elicit a behavioural reaction in conspecific males. In agreement with that assumption, we did not detect any p-anisaldehyde in MTBE extracts of body parts from dissected individuals that had been kept in captivity under laboratory conditions for 2–15 h before analysis. However, the compound was found in samples of volatiles collected directly in the natural habitat of *C. ciliata* and when using living insects within 2–22 h of capture in the habitat where they were caught. The less volatile compounds methyl p-anisate and p-methoxybenzoic acid were detected in the extracts from the cuticle of the body parts with an abundance of elliptical glands (2nd to 8th abdominal segments in males). The same compounds were not detected in extracts of body parts without glands, which suggests that these compounds are either precursors or oxidative by-products involved in production of p-anisaldehyde. Hypothetically, it is possible that p-anisaldehyde is produced by reduction of p-methoxybenzoic acid, which might be initially formed from the methyl ester (i.e., methyl p-anisate). Alternatively, both p-methoxybenzoic and methyl p-anisate, might be reduced directly to yield p-anisaldehyde, which means that the insect would store p-methoxybenzoic acid and methyl p-anisate as less volatile precursors that are ready for the enzymatic production of the active and more volatile p-anisaldehyde when ecologically needed (John Pickett, Cardiff University, UK, pers. comm.). This picture is analogous to earlier findings on the Nasonov pheromone of the honeybee *Apis mellifera* L. (Hymenoptera: Apidae) and the mechanism involved in regulation of the pheromone production of that species (Pickett et al. 1980, 1981). In the cited studies, the authors elucidated an enzymatic mechanism for the formation of highly volatile pheromone components in specific glands based on less volatile chemical precursors. Such a regulation mechanism enables pheromone production that is closely related to the physiological needs of the insect in question (Pickett et al. 1981). Another hypothesis is that p-anisaldehyde that is not in use might be deactivated by enzymatic oxidation. This would initially yield p-methoxybenzoic acid, which could be converted by esterification to the methyl ester (i.e., methyl p-anisate, which is physiologically less irritating than the free acid) for storage and recycling. Further studies are needed to verify the semiochemical pathway in this context.

P-anisaldehyde is a common plant volatile (Knudsen et al. 2006) that is known to be a semiochemical signal for a number of arthropod species (El-Sayed 2019; Morgan and Crumb 1928). For example, p-anisaldehyde has been found to be attractive to several species of thrips (Thysanoptera: Thriphidae) (Hollister et al. 1995; Kirk 1985; Koschier et al. 2000) and the apple fruit moth *Argyresthia conjugella* Zeller (Lepidoptera: Argyresthiidae) (Bengtsson et al. 2006; Knudsen et al. 2017). Also, in the varied carpet beetle *Anthrenus verbasci* L. (Coleoptera: Dermestidae), p-anisaldehyde has been reported to be a potent attractant for both sexes, and for males is nearly as attractive as the female sex pheromone (Imai et al. 2002). In addition, p-anisaldehyde has been identified as a minor component of the foraging recruitment pheromone of the bumblebee *Bombus terrestris* L. (Hymenoptera: Apidae) (Granero et al. 2005). Repellent effects of p-anisaldehyde have also been recorded for species such as the lone star tick *Amblyomma americanum* L. (Acari: Ixodidae) (Showler and Harlien 2018a), the horn fly *Haematobia irritans irritans* L. (Showler and Harlien 2018b), and the house fly *Musca domestica* L. (Diptera: Muscidae) (Showler and Harlien 2019).

In our study, p-anisaldehyde was a powerful attractant for *C. ciliata* males provided that a solid population of this species was existing in the trapping area. Surprisingly, no conspecific females were captured in our field-trapping experiments. Similarly, no females were caught by sweep netting in the surroundings of the traps. Previous investigations have shown attraction of males to the male-produced compound (1R*,2S*,5R*,8R*)-iridodial in *Chrysopa oculata* (Zhang et al. 2004) and to a closely related compound in *C. nigricornis* (Zhang et al. 2006a). Furthermore, the compound iridodial was found as a highly powerful attractant for males of *C. septempunctata* (Zhang et al. 2006b). Although very seldom conspecific females were caught in the traps, often females were observed surrounding the traps, but not entering (Chauhan et al. 2007). The authors presumed either that females use a male-produced pheromone for long-range orientation, while calling males acoustically at a close range, or that females require male-produced acoustical or vibrational cues for close-range orientation (Aldrich and Zhang 2016; Chauhan et al. 2007; Henry et al. 2013; Zhang et al. 2004, 2006a, b). In contrast, in our assessments, *Chrysotropia ciliata* females were never observed in the surroundings of the traps. We found females of this arboreal lacewing species only in the crowns of the trees (height 7–10 m), although the traps were located in ground-level forest vegetation (height 1–2 m). Thus, it is possible that placing the traps at the lower level of the tree vegetation might be why no females were caught. However, while trapping *C. ciliata* for further analyses using different trapping methods at different levels of the tree, we observed mainly females and only single males high up in the canopy, which suggests different niche preferences of males and females within the same tree. In studies conducted in other European forests, most specimens of this species were also trapped at canopy levels at a height of 5–15 m (**Makarkin and Ruchin**^2019^**;** Saure and Kielhorn 1993) and even higher at 15–35 m (Gruppe 2008; Gruppe and Schubert 2001; Nielsen 1977). Different niche preferences within the same tree have been noted for individuals of the same species depending on developmental stage, and this includes *C. ciliata* (Gepp 1987). The concentration of *C. ciliata* females at higher canopy levels may be explained by the need for honeydew consumption (ripening nutrition) for proper egg production and microhabitats for their larval prey populations for suitable egg-laying. *C. ciliata* females can find homopteran populations providing these conditions for successful reproduction at higher canopy levels in shoot ends and younger leaves. In addition, *C. ciliata* males reside in treetops, as do the females, and the males only move to the ground level due to the high attraction of p-anisaldehyde.

We also observed that *C. ciliata* males attracted to p-anisaldehyde attempted to either bite the septum containing p-anisaldehyde or bite each other. Such peculiar behaviour might be interpreted as competition for females, with the septa representing an alpha male releasing a large amount of p-anisaldehyde. Similarly, it has been reported that males of *Chrysopa* spp. eat the native silver vine *Actinidia polygama* Siebold and Zucc. (Actinidiaceae), because such plants contain compounds that are stereochemically similar to the lacewing pheromone (Aldrich and Zhang 2016; Aldrich et al. 2016; Hyeon et al. 1968; Yoshihira et al. 1978). Aldrich et al. (2016) hypothesized that this specific male behaviour in *Chrysopa* might be motivated to obtain substances from prey or plants as precursors for production of their pheromones.

The function of p-anisaldehyde in context of the ecology of *C. ciliata* is cryptic and we can only speculate here. Based on the strong male-male attraction, the observed male-male aggression and the fact that females seem to not be attracted at all, p-anisaldehyde might have a function as male-specific alarm, aggression or aggregation pheromone, perhaps as part of a mating strategy controlling male population density and thus, benefit the species by secure resources (Stevenson and Rillich 2019; Verheggen et al. 2010; Wang and Anderson 2010). Studies on similar phenomena are rare. As an example, a study on *Thaumastocoris peregrinus* Carpintero and Dellapé (Heteroptera: Thaumastocoridae) found a male-produced volatile compound, namely 3-methylbut-2-enyl butanoate, attractive to conspecific males only. The authors suggested a male specific aggregation pheromone as biological function for this compound (González et al. 2012). In another study the male specific compounds hexyl butyrate and (*E*)-2-hexenyl butyrate have been found in *Phytocoris difficilis* Knight (Hemiptera: Miridae). These compounds have resulted in an interruption of attraction of males to the female produced sex pheromone. The authors suggested the compounds to be anti-sex pheromones involved in a mate guarding strategy (Zhang and Aldrich 2003).

It has been observed in this study and previous investigations, that *C. ciliata* occurred regional in high densities, often aggregating in a group of trees or single trees, and here mass occurrences of this species have been noted in particular periods (Aspöck et al. 1980; Duelli et al. 2002; Gepp 1977; Nielsen 1977; Saure and Kielhorn 1993; Szentkirályi 2001c). Based on this knowledge we can hypothesize that in a given habitat, *C. ciliata* females select the trees for mating and oviposition as a good resource for their offspring and then calling conspecific females and males by vibrational and/or semiochemical signals to these particular spots. Thus, it is possible that if a male is approaching a region with a group of females he releases p-anisaldehyde to give an additional signal to conspecific males that this is a good place for mating. The males receiving p-anisaldehyde in moderate concentrations, as found in our field headspace collection from live males, get attracted to the same spot (long-range attraction) and will then locate a female for mating by female-specific signals (close-range attraction). If the males get attracted to high p-anisaldehyde concentrations, but not receiving female signals at the p-anisaldehyde source and/or facing many males there, the male behaviour might switch to aggression, as observed in some of our field trapping experiments. In such a hypothetical scenario, p-anisaldehyde acts as male-male signal within a mating strategy controlling male population density to ensure mating efficiency.

Additional studies in the laboratory and field are needed to test the extent to which these hypotheses might apply to *Chrysotropia ciliata* as well as to fully understand the ecology of this in many ways cryptic species. Beside providing further knowledge of the chemical ecology of this chrysopid*,* additional trapping studies based on the new chemical attractant of *C. ciliata* males identified in our investigation would enable a more accurate spatial and temporal monitoring of the seasonality of this species. Perhaps such surveillance could prove to be important in the future, because the population dynamics of hygrophilous *C. ciliata* may be a good indicator of any tendencies towards forest aridification caused by climatic changes.
